# Trends in commercial complementary food in Romanian infants–a cross-sectional study

**DOI:** 10.3389/fnut.2025.1622842

**Published:** 2025-09-18

**Authors:** Elena Roxana Matran, Andra-Mihaela Diaconu, Cristina Adriana Becheanu

**Affiliations:** ^1^Department of Paediatrics, “Carol Davila” University of Medicine and Pharmacy, Bucharest, Romania; ^2^Grigore Alexandrescu Emergency Hospital for Children, Bucharest, Romania

**Keywords:** infant, complementary feeding, CF, commercial baby food products, nutrition

## Abstract

**Background/objectives:**

Good feeding practices beginning early in life and are crucial for preventing all forms of malnutrition and non-communicable diseases. This time frame encompasses the delicate phase of complementary feeding, which traditionally involved homemade meals. The use of commercial complementary foods (CCF) began more than a century ago and represents a convenient alternative. We aim to outline both the profile of CCF consumers while accurately describe CCF dietary patterns.

**Materials and methods:**

We conducted a cross-sectional study analysing a final cohort of 75 infants 6–12 months admitted for acute illnesses to the Pediatrics Department of the “Grigore Alexandrescu” Emergency Hospital for Children in Bucharest, Romania, from June 2024 to December 2024. The mothers were requested to complete a two-section questionnaire focusing specifically on the utilization of commercial baby food products.

**Results:**

Eighty percent of the study population consumed at least once a CCF product, with a median [IQR] age at first administration at 6 months [5.25–7]. The CCF products were divided in 6 categories: milk-based products, cereals, pseudocereals, fruit jars/pouches, vegetables puree and meat jars and biscuits and pastas (flour-based products) similar to the one from European Commission. The most frequently given were biscuits and pasta. CCF consumption was not overall influenced by family income or educational level, except the pseudocereals consumption. Among the most utilized vegetables were sweet potatoes, carrots, zucchini, among the fruits were apples and banana and chicken-meat was the most offered. Overall perception of mothers on CCF was favorable, within the motivations and advantages of using them being their diversity and convenience.

**Conclusion:**

CCF are intensely utilized in our country. Regarding the composition of these products, there is a combination between traditions and new dietary tendencies. Longitudinal, further studies, are necessary to characterize the long-term effects of this feeding pattern.

## 1 Introduction

According to the World Health Organization (WHO), an optimal growth and development while preventing all forms of malnutrition associated with feeding practices are obtained through good nutrition practices beginning in infancy (1).

Complementary feeding (CF) is defined in a report for the European Commission by the European Food SafetyAuthority (EFSA) Panel on Nutrition, Novel Foods and Food Allergens as the time frame when complementary foods (CFs) are administered together with breast-milk and/or formula, water or vitamins and comprise beverages, pureed-foods for spoon-feeding, more lumpier foods or finger food, prepared in households or commercially (2). European Society of Gastroenterology, Hepatology and Nutrition (ESPGHAN) states that CF “should include all solid and liquid foods other than breast milk or infant formula” (3). Contradictory data exist regarding the appropriate age for introducing CF. The WHO recommends a fixed age of 6 months (180 days) (4), while European, Asia-Pacific, North American, Latin American, and Pan-Arab professional societies support the possibility of initiating CF as early as 17 weeks (beginning of the fifth month of life) (5). The current ESPGHAN guideline on CF, recommends rather a specific time frame for initiating CF, between 17 and 26 weeks and advocate for the use of age-appropriate foods with suitable consistency, administered in a manner appropriate to the infant’s age and development (3).

Until 1989, under Romania’s communist regime, food was rationed and parental leave was short. Emil Căpraru’s 1974 book “Mama și copilul” recommended starting complementary foods at 3 months. Early diversification involved fruit juices and grated apples with sugar syrup, increasing in quantity and thickness over time. Malnourished infants received added biscuits or bread crumbs. Bananas were limited by availability despite recommendations. Later, vegetables thickened with butter or flour were introduced, along with milk-thickened vegetable and fruit powders. Commercial baby food jars were costly and promoted from three to 4 months when available (6).

The definition of commercial infant food products implies pre-packaged, “ready-to-serve” food items specifically formulated for this age group. These products are manufactured by specialized infant food companies and require minimal thermal preparation or heating prior to consumption. In comparison, homemade foods are prepared in households by the caregivers, with mainly fresh ingredients (7). Commercial complementary food (CCF) is intended for children aged 4 months to 36 months (8). Despite this, CCF differ in composition according to age segment (infants vs. toddlers). Furthermore, studies indicate that errors related to the nutritional content of CCF are more frequent and more commonly reported in toddlers compared to infants (9).

While some families still prefer to provide CF using homemade meals, an increasing number of parents are opting for CCF for their infants. This shift is evidenced by the ongoing growth and development of the CCF industry, valued at $ 82.84 billion in 2024 (10). In 2025, Romania ranked 12th among the 27 European Union member states in terms of gross domestic product (GDP), classifying it as a developing economy (11) Despite the widespread availability and early introduction of CCF across Central and Eastern Europe — often before the recommended age of 6 months (12)—such products remain by comparison less established in Romania. Unlike other European countries where commercial baby foods have been accessible to affluent families since as early as 1869 (13), Romania’s CCF market is comparatively new and underdeveloped. Commercial baby food was a niche market in Romania at the end of the last decade, with low consumption attributed both to price sensitivity and preference for homemade foods (14). Romanian consumers are increasingly prioritizing health and nutrition in their baby food choices, driving demand for organic, locally sourced, and convenient products such as ready-to-eat and on-the-go options. This trend fuels the growth of the baby food market, which reflects a blend of traditional and modern dietary preferences. While familiar ingredients like carrots and dairy remain prevalent, newer items such as imported sweet potatoes and pseudocereals are gaining popularity. This combination shows Romanian consumers’ desire for nutritious, convenient products that honor cultural food traditions while embracing evolving nutritional trends (15).

Romania is actively aligning with and occasionally exceeding EU regulations on processed baby foods, demonstrating commitment to improved nutritional quality and transparent claims (16). Additionally, Romania has introduced national measures surpassing EU standards, including proposed bans on infant formula promotion up to 2 years and mandatory warning labels supported by scientific evidence (17).

Hajdú et al. address CCF consumption across infants and toddlers (1–3 years) in Romania and Hungary. Infant and toddler feeding differ significantly due to age-specific dietary needs. Despite geographic and regulatory similarities, the two countries differ in cultural values and feeding practices. Romanian mothers use commercial jarred baby foods much less frequently than Hungarian mothers—about one-third versus nearly 90%—primarily due to a preference for homemade foods and distrust of commercial products. Furthermore, Romania’s market is dominated by imported brands, unlike Hungary where local brands are available. Romanian mothers emphasize trust over price and are more likely to use CCF if living in urban areas, being older, and having fewer children. Conversely, Hungarian mothers prioritize convenience and dietary variety, pay closer attention to ingredient and allergen information, while Romanian mothers often struggle with label clarity and seek better transparency (18).

Romanian consumers—especially in urban areas—regularly read food labels and are particularly attentive to information about additives, shelf life, and known risks. A high emphasis was found on nutritional facts and health claims, and there is documented woriness toward perceived “artificial” or unclear ingredients, both in local and imported products (19). Moreover, Draghici et al. (20), highlight a preference among Romanian consumers for organic products, motivated by mistrust of conventional food manufacturing processes, uncertainty about additives, and a demand for greater ingredient.

Regardless of the options chosen by parents, feeding practices during CF are crucial for a child’s growth and development. This is a critical time to implement healthy eating behaviors that can have a lasting impact throughout life (21) and parents have the primary responsibility of this.

To increase the rigor of the CCF, the International Code of Marketing of Breast-Milk Substitutes was implemented in 1981, updated numerous times by World Health Assembly resolutions.

Thus, numerous articles emphasize that there is a wealth of information available about the content of CCF. There are also rising concerns regarding the increasing consumption of CCF and the inappropriate reasons behind parents’ choices. This is particularly surprising given that, in our fast-paced era, parents are merely one click away from discovering whether a particular product, based on its nutritional label, is suitable for their child’s diet.

To our knowledge, this is the first Romanian study focusing on CCF that concurrently examines the detailed dietary preferences of families, the maternal and child profile.

The aim of this study was to focus on consumers of CFF, to identify trends related to CFF, determine which products were most commonly used and how often they are consumed. Additionally, the study described patterns of dietary attitudes within families, related to factors such as education, income and socioeconomic status.

## 2 Materials and methods

A cross-sectional study was conducted utilizing a convenience sample of 75 infants admitted to the Pediatrics Department of the “Grigore Alexandrescu” Emergency Hospital for Children in Bucharest, Romania. The study period extended from June 2024 to December 2024, and participants were admitted for various acute, non-complicated respiratory or gastrointestinal conditions. Inclusion criteria were as follows: (1) age between 6 and 12 months, (2) initiation of CF, and (3) obtained consent from the legal guardian. The accompanying parent, who in all cases (100%) was the mother, was requested to complete a two-section questionnaire. The first section contained information on: child demographics, feeding patterns, nutritional status, maternal educational level and mean monthly family income. The second section focused specifically on the utilization of commercial baby food products.

### 2.1 Patient data

Demographic and clinical information were collected from the child’s history. Additionally, the following parameters were documented: Birth order (first born, second born, ≥3^rd^ child), birth weight (g), feeding pattern in the first 6 months, age at initiation of complementary feeding (CF, months), nutritional status at enrolment (Z score for weight-for-age). Feeding patterns were categorized as: breastfed-only, mixed feeding (minimum 8 weeks of breast milk) and exclusive formula-fed. Anthropometric measurements, specifically actual weight (g), were obtained. Nutritional status was evaluated by converting weight-for-age to standardized Z-scores and classified according to World Health Organization (WHO) references for children aged 0–2 years (22). Additional data included: mother’s age at the infant’s enrolment (years), mother’s level of education, employment status and monthly family income (RON/family).

The monthly family income was stratified into three categories based on the Romanian national minimum wage which was 3,300 RON (approximately 670 euros) in June 2024. The income stratification was delineated into three distinct categories: < 3,300 RON, 3300–6,600 RON, and ≥ 6,600 RON. The rationale for this tripartite division was to capture with greater precision the middle-income layer, which constitutes the majority in our country, while also delineating the above-average income group.

According to the level of education, the patients were divided also in three categories, based on maternal education: no formal education group, secondary education group (high school) and the higher education group (university degree and post-university studies).

### 2.2 Product data

The CCF products were divided in 6 categories: milk-based products (yogurt with or without fruits, puddings), cereals (containing gluten or gluten-free), pseudocereals (buckwheat, quinoa, amaranth), fruit jars/pouches, vegetables puree and meat (chicken, turkey, fish, beef) jars and biscuits and pastas (flour-based products) similar to the one from European Commission (23). Furthermore, we included the category of pseudocereals due to the recent trends we have observed in infant nutrition. Questions about age of first administration (months), frequency of administration (1–2 times/week, 3–5 times/week, ≥ 6 times/week) and type of utilized product (both first time and afterwards) were included.

Additional queries about the overall maternal impression on the CCF products, primary reason for use (meals diversity, nutritional content, good palatability, trusted brand, advertising, price) and avoidance (extended shelf life, ingredient quality, sugar and salt content, others), information sources for product selection (family/friends, medical personnel), initial source of product awareness (social media, family and friends, advertising in supermarkets or pharmacies, medical personnel), perceived advantages (convenience, good palatability, baby soothing, diversity)/disadvantages (extended shelf-life, sugar and salt content, gastrointestinal disturbances associated with their use, homemade food-refusal, price or no disadvantages) of CCF product use, acquisition sources (pharmacies, supermarkets or both), context of utilization (at home or on-the-go), attention to the product nutritional labels.

To ensure thoughtful responses, the questionnaire was returned after a minimum of 24 h, allowing sufficient time for consideration and potentially increasing the accuracy and completeness of the collected data.

The study was approved by the Ethics Committee of “Grigore Alexandrescu” Emergency Children’s Hospital (#35/07.10.2024) and conducted in accordance with the Declaration of Helsinki. Patient’s confidentiality and privacy were maintained throughout the study.

### 2.3 Statistical analysis

Data were analyzed using SPSS v26 (Chicago, IL, United States). Descriptive statistics were computed for both continuous and categorical variables. Continuous variables were evaluated for normality using the Kolmogorov–Smirnov test. For continuous variables, the mean/ median with standard deviations (SD) or interquartile range (IQR) as appropriate were used, regarding their distribution. To analyze the association between categorical variables, we employed either chi-square test or Fisher’s exact test, depending on the sample size and expected cell frequencies and *φ* coefficient was used to measure the strength of the correlations. A Chi-square goodness of fit test was performed to evaluate the distribution of categorical outcomes in our sample. To address the limitations of small sample sizes and low expected frequencies in our categorical data analysis, we employed Monte Carlo simulation method. Pearson correlation was used to evaluate the associations between continuous variables that demonstrated normal distribution. To compare means and medians of continuous variables between more than two groups, one-way Anova was used for the normally distributed data and the Kruskal–Wallis one-way for the non-normally distributed data. Independent-T-test was used to compare means of normally distributed variables between two groups. *Post hoc* analysis was performed for ANOVA and Kruskal–Wallis, when significant differences were found between groups. A *p* value < 0.05 was considered as the threshold for statistical significance.

## 3 Results

Eighty-nine patients were recruited initially. Of them, for 5 patients we did not obtain consent from their mothers. Additionally, 9 patients were excluded due to pre-existing conditions like food-allergies or severe underweight status of different aetiologies that significantly altered their normal feeding patterns. After applying these exclusion criteria, a total of 75 patients were enrolled, 46 (61.3%) boys. The median (IQR) age at inclusion was 8 months (6–10). Median [IQR] age at initiation of CF was 6 months (5, 6) and mean (±SD) mother’s age was 29 years (±6.4). Most infants [*n* = 57, (76%)] had normal nutritional status or overweight. Sixty (80%) infants were administered at least once a CCF product, *p* < 0.001. First administered products were in order of their distribution: biscuits and pastas (combined in “flour-based products”) in 16 infants (26.7%), fruits puree in 14 infants (23.3%), cereals (including pseudocereals) in 12 infants (20%) and yogurt and vegetables/vegetables with meat jars, each in 9 infants (15%), statistically non-significant. Median [IQR] age at first administration of a CCF product is 6 months [5.25–7] (Table 1).

**Table 1:** Characteristics of the population.

Characteristics	CCF positive *n* = 60 (80)	CCF negative *n* = 15 (20)	*p*-value
Sex [*n* (%)]			0.506
Male	38 (63.3%)	8 (53.3)	
Female	22 (36.7%)	7 (46.7)	
Median age at inclusion, months [IQR]	8 [7–10]	7 [6–9]	0.451
Median age at CF, months [IQR]	6 [5–6]	6 [6–6]	0.275
Mean mother’s age, years [SD]	28.6 [±6.6]	30.7 (±6.3)	0.273
Residence [*n* (%)]			0.05
Urban	38 (63.3)	10 (66.7)	
Rural	22 (36.7)	5 (33.3)	
Birth order [*n* (%)]			0.167
1st	32 (53.3)	9 (60)	
2nd	16 (26.7)	3 (20)	
≥3rd	12 (20)	3 (20)	
Nutritional status [*n* (%)]			0.897
Healthy weight	40 (66.7)	11 (73.3)	
Under weight	15 (25)	3 (20)	
Overweight/obesity	5 (8.3)	1 (6.7)	
Feeding type [*n* (%)]			0.791
Formula-fed	19 (31.7)	6 (40)	
Mixed-feeding	25 (41.7)	6 (40)	
Breast-fed	16 (26.6)	3 (20)	
Mother’s educational level [*n* (%)]			0.686
Unschooled	4 (6.7)	2 (13.3)	
Medium level	30 (50)	7 (46.7)	
Higher education	26 (43.3)	6 (40)	
Income level, RON/month [*n* (%)]			0.415
Low, ≤ 3,600	22 (36.7%)	5 (33/3)	
Medium, 3,601–6,600	16 (26.7)	2 (13.3)	
High, ≥ 6,601	22 (36.6)	8 (53.3)	
Mother’s employment			0.336
No	8 (53.3)	7 (46.7)	
Yes	40 (66.7)	20 (33.3)	

CCF, commercial baby food; CF, complementary feeding; IQR, interquartile range; RON, Romanian currency; SD, standard deviation.

**Figure 1:**
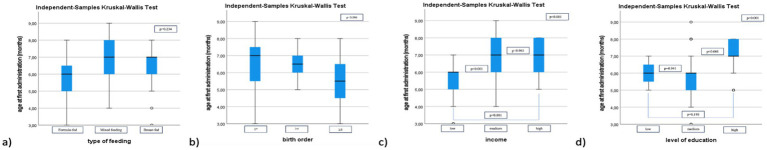
Box-plot representation of “age at first administration of CCF” distribution according to: **(a)** type of feeding, **(b)** birth order, **(c)** income, **(d)** level of education.

The residence and gender did not significantly influence this parameter nor the feeding type in the first 6 months and the birth order did; however, a notable trend was observed with respect to birth order, suggesting a tendency for earlier CCF product administration with increasing birth order, although this trend did not reach statistical significance. Despite comparable median values across the three subgroups within both income and education level categories, the statistical analysis revealed significant overall differences (*p* < 0.001 for both variables), indicating that infants from high income families and with highly-educated mothers, have older ages at introduction of CCF products. This discrepancy is presumably attributable to variations in the distribution of values within each subgroup, rather than differences in central tendency (Figure 1). Moreover, no statistically significant correlation was observed between maternal age and the age at initial administration of CCF products. The main motivations and concerns regarding the use of CCF are indicated in Table 2.

**Table 2:** Motivations and reluctance causes toward commercial baby food products.

Main motivations (*n* = 60)	[*n* (%)]	*p*-value	Causes of reluctance (*n* = 15)	[*n* (%)]	*p*-value
Diversity	11 (18.3)		Extended shelf life	4 (26.7)	
Nutritive	10 (16.7)		Doubtful quality	3 (20)	
Easily accepted (good palatability)	25 (41.7)	<0.001	Sugar/salt content	1 (6.7)	0.186
Trusted brand	8 (13.3)		Other	7 (46.6)	
Advertising Price	5 (8.3) 1 (1.7)				

Forty-five (75%) of the mothers read the nutritional label when purchasing a CCF product, while acquisition sources revealed no statistically significant differences in preferences among the studied group: from the pharmacy, 12 (20%), supermarket, 23 (38.2%) and from both, in 25 (41.7%) of cases. The source for product awareness comes mostly from advertising for 22 (36.7%) subjects, followed by family/friends in 19 (31.7%) subjects and social media in 12 (20%) subjects. Conversely, healthcare professionals demonstrated the lowest probability of disseminating product-related information (7, 11.6%), but without statistical significance. Meanwhile, for advice on the use of these products, mothers primarily rely on their own knowledge or that obtained from medical personnel (28, 46.7%, respectively, 20, 33.3%); guidance from peers and relatives being the least frequently employed (12, 20%), *p* = 0.04. The administration of CCF products to infants showed no statistically significant difference between home and out-of-home settings (27, 45% vs. 33, 55%). Analysis revealed no statistically significant association between the utilization of a specific CCF product and socioeconomic indicators, like income and level of educational (Table 3), except for the use of pseudocereals which demonstrated moderate association with families with high income and elevated level of mother’s education (*p* = 0.011, *φ* = 0.399, *p* = 0.008 respectively, φ = 0.406).

**Table 3:** Associations between the categories of commercial complementary food use and income and educational level.

	Income				Education			
Type of CCF	Low	Medium	High	*p*-value	Unschooled	Medium	High	*p*-value
Cereals [*n*, (%)] No [*n* = 27, (45)]	13 (48.1)	5 (18.5)	9 (33.4)	0.208	2 (7.4)	15 (55.6)	10 (37)	0.663
Yes [*n* = 33, (55)]	9 (27.3)	11 (33.3)	13 (39.4)		2 (6)	15 (45.5)	16 (48.5)	
Pseudocereals [*n*, (%)] No [*n* = 48, (80)]	20 (41.7)	15 (31.3)	13 (27)	0.011	4 (8.3)	28 (58.3)	16 (33.4)	0.008
Yes [*n* = 12, (20)]	2 (16.7)	1 (8.3)	9 (75)		0	2 (16.7)	10 (83.3)	
Fruit purées [*n*, (%)] No [*n* = 35, (58.3)]	15 (42.9)	8 (22.8)	12 (34.3)	0.481	3 (8.6)	19 (54.3)	13 (37.1)	0.511
Yes [*n* = 25, (41.7)]	7 (28)	8 (32)	10 (40)		1 (4)	11 (44)	13 (52)	
Vegetables/vegetables with meat jars [*n*, (%)] No [*n* = 34, (56.7)]	15 (44.1)	9 (26.5)	10 (29.4)	0.314	3 (8.8)	18 (52.9)	13 (38.2)	0.652
Yes [*n* = 26, (43.3)]	7 (27)	7 (27)	12 (46)		1 (3.9)	12 (46,1)	13 (50)	
“Flour-based products” (biscuits and pastas) [*n*, (%)] No [*n* = 21, (35)]	8 (38.1)	5 (23.8)	8 (38.1)	0.935	0	13 (62)	8 (38)	0.252
Yes [*n* = 39, (65)]	14 (35.9)	11 (28.2)	14 (35.9)		4 (10.3)	17 (43.6)	18 (46.1)	
Dairy based products [*n*, (%)] No [*n* = 22, (36.7)]	7 (31.8)	6 (27.3)	9 (40.9)	0.844	2 (9)	10 (45.5)	10 (45.5)	0.769
Yes [*n* = 38, (63.3)]	15 (39.5)	10 (26.3)	13 (34.2)		2 (5.3)	20 (52.6)	16 (42.1)	

CCF, commercial baby food.

Regarding the consumption of specific ingredients in CCF products, across all categories, mothers predominantly opt for a combination of ingredients. However, certain particularities are noteworthy. Although gluten-containing cereals are the most used, almost a quarter of infants receive gluten-free products. Among pseudocereals, quinoa and amaranth are the most frequently utilized. In the fruit category, apples, bananas, and pears are predominant. Surprisingly, for our country, sweet potato emerges as the most preferred vegetable, followed by indigenous vegetables such as carrots and zucchini. With respect to meat consumption, chicken is by far the most frequently administered, followed by beef and turkey, with fish being the least consumed protein source (Table 4).

**Table 4:** Consumption of specific ingredients in commercial baby food, among study population (*n* = 60).

Ingredients in CCF	Types	Utilization [*n*, (%)]*
Cereals *N* = 33	Gluten containing	21 (63.6)
	Gluten-free	8 (24.3)
	Both	4 (12.1)
Pseudocereals *n* = 12	Mix	8 (66.7)
	Quinoa	4 (33.3)
	Amaranth	2 (16.7)
	Buckwheat	0 -
Fruits *n* = 25	Mix	17 (68)
	Banana	5 (20)
	Apples	7 (28)
	Pears	4 (16)
Vegetables *n* = 26	Mix	24 (92.3)
	Sweet potatoes	4 (15.4)
	Carrots	3 (11.5)
	Zucchini	2 (7.7)
Meat *n* = 26	Chicken	22 (84.7)
	Beef	10 (38.5)
	Turkey	9 (34.7)
	Fish	7 (26.9)

CCF, commercial baby food. *The cumulative percentage may exceed 100% as subjects could be classified into multiple categories simultaneously.

Analysis of consumption frequency revealed a statistically significant difference in the utilization of various product categories. In descending order of prevalence, the most frequently consumed products were: flour-based products (biscuits and pastas), dairy-based products, cereals, vegetable/vegetable-meat combination jars, fruit purées, and pseudocereals (Table 5).

**Table 5:** Consumption frequency for each category of commercial complementary food.

	Frequency			
Categories of CCF product*	1–2 times/week [*n*, %]	3–5 times/week [*n*, %]	6–7 times/week [*n*, %]	*p*-value
Cereals (*n* = 33, 55%)	22 (66.7)	4 (12.1)	7 (21.2)	<0.001
Pseudocereals (*n* = 12, 20%)	7 (58.3)	3 (25)	2 (16.7)	0.174
Fruits puree (*n* = 25, 58.3%)	15 (60)	4 (16)	6 (24)	0.016
Vegetables/vegetables with meet jars (*n* = 26, 43.3%)	15 (57.7)	7 (27)	4 (15.3)	0.024
Flour-based products (*n* = 39, 65%)	22 (56.4)	8 (20.5)	9 (23.1)	0.009
Dairy based-products (*n* = 38, 63.3)	23 (60.5)	8 (21.1)	7 (18.4)	0.003

Perceived advantages and disadvantages associated with CCF are noted in Table 6.

**Table 6:** Perceived advantages and disadvantages associated with commercial baby food products consumption.

Advantages (*n* = 60)	[*n* (%)]	*p*-value	Disadvantages (*n* = 15)	[*n* (%)]	*p*-value
Convenience	38 (63.3)	*p* < 0.001	None	32 (53.3)	*p* < 0.001
			Doubtful quality	12 (20)	
			Extended shelf-life	8 (13.3)	
Easily accepted (good palatability)	18 (30)		Sugar/salt content	3 (5)	
Diversity	3 (5)		Won’t accept home-made food	3 (5)	
			Price	1 (1.67)	
			Gastrointestinal disturbances	1 (1.67)	

## 4 Discussion

To our knowledge, this is the first study to present detailed data on the consumption of CCF among Romanian infants. Several authors from our country have discussed various aspects of infant feeding practices; however, they have not addressed this particular aspect. For instance, with respect to breastfeeding, Cozma-Petrut et al. conducted a study in 2019 among 1,399 mothers of children aged 0–23 months, which revealed a positive trend in breastfeeding rates in Romania over the past decade. The study found that nearly all mothers (95.7%) breastfed their child at least once and the exclusive breastfeeding rate was 46.7% (24). In our study, 41.3% of mothers offered their infant a mixed-type of feeding, and 25% of them went exclusive breastfeeding their child. However, the number of participants in our study is significantly smaller, and the age segment analyzed is much narrower. Furthermore, we defined in our study “mixed-feeding” as in formula-feeding with at least 8 weeks of breast-feeding. As regards CF, Becheanu et al. reported in a 2018 longitudinal study that 85.6% of infants were correctly introduced to CF in terms of age (between 4 and 6 months) and foods, while 8.9% were weaned prematurely, and 5.5% experienced this transition after 7 months of age (25). Similarly, in our cohort, CF was mostly initiated at the age recommended by guidelines, with a median start at 6 months.

The discussions surrounding complementary feeding practices, specifically the comparison between commercial and home-made infant foods, emerged over four decades ago and has been since then a subject of ongoing debate in the field of pediatric nutrition although at the same time, validating CCF as nutritionally equivalent to home-prepared meals (26).

In selecting CFs for their infants, parents may opt for homemade meals or CCF products or a combination of them. This decision-making process involves weighing different factors related to nutritional content, convenience, and cultural preferences. Contrary to the specialized literature which reports that nearly one-third (33.3%) of consumers read the label on infant foods (27), we identified interest in the nutritional content of CCF at a significantly higher percentage.

Over time, the nutritional composition of diet has been the primary focus of numerous articles on CCF, as the nutritional intake throughout childhood, adolescence, and early adulthood has significant implications for overall health and disease risk. The most comprehensive study in Europe that enables the longitudinal assessment of dietary patterns and nutritional trends in children is the DONALD (Dortmund Nutritional and Anthropometric Longitudinally Designed) study, established over 35 years ago as an open-cohort study in Dortmund, Germany. The DONALD study provides invaluable insights into habitual nutrient and food intake across different developmental stages and its findings contribute to public health initiatives by informing national and international nutrition guidelines and policy recommendations. Since 2005, the study has also extended its data collection to include participants in adulthood, facilitating the investigation of the long-term impact of nutrition and lifestyle factors during life stages that are considered critical for the development of chronic diseases (28).

Hilbig et al. also compared CCF and home-made meals by recruiting 396 participants from the German DONALD study. Their findings indicated that, aside from a few differences such as higher sodium content in commercial savory meals for older infants, lower fat content in commercial savory and cereal–fruit meals, and added sugar in some commercial dairy–fruit meals—there were no significant nutritional inadequacies between commercial and homemade complementary meals (29). On the contrary, Garcia et al. indicated the nutritional superiority of homemade meals, with the exception of ruscks and biscuits which are higher in iron and calcium than their homemade alternative, but high on sugar (30).

However, one of the most debated topics regarding CCF remains its sugar content. In a study conducted in 2019, Hutchinson et al. aimed to determine whether CCF marketed in Europe (for children up to 36 months old) contained high levels of sugar and to provide recommendations for updating European regulations and guidelines. The research revealed that, in each of the 10 studied European countries (United Kingdom, Denmark, Spain, Italy, Malta, Hungary, Norway, Portugal, Estonia, and Slovenia), approximately one-third of the total energy in CCF came from sugar. This level is considered high and contradicts the WHO’s existing recommendation to limit free sugars in foods for this age group (31).

Nevertheless, a pivotal aspect of CCF is the parental opinion about this type of feeding. In recent years, researchers have focused on the typology of parents who choose whether or not to administer such a product to their child in order to understand their motivation and, respectively, their reluctance regarding CCF. Extensive industrialization, the tendency of families to move to urban areas, the proliferation of supermarkets, the introduction of infant food products in these stores (making them much more accessible), rising incomes, and repeated exposure to various marketing strategies have led some parents to choose CCF for their infants, both for light meals and main meals. One major determinant of CCF use is reported to be the consumer confidence in the food industry which exhibits a significant and positive correlation with the utilization of CCF (7).

In the United Kingdom, an Infant Feeding Survey was initiated in 1975, providing data on feeding patterns at five-year intervals. The most recent available data dates back to 2010. One of the key survey questions assessed the dietary intake of infants during the preceding 24 h. Findings indicate that, among infants aged 4 to 6 months, CCF were the predominant source of nutrition compared to home-prepared meals. Conversely, in infants aged 8 to 10 months, there was a higher prevalence of home-prepared food consumption, with a reduced reliance on CCF (32).

A report by Euromonitor titled “Baby Food in Romania” indicates that retail sales of baby food in Romania, reached 540 million RON in 2024, representing a 7% increase compared to 2023, despite declining sales volume due to higher prices and the migration of young families, thus being significantly impacted by the high cost of living (33). Usage of these products may be influenced by maternal age, method of feeding in the first 6 months of life, presence of other children in the household, region and food availability (34).

Similar to a report from West Africa that comprised a significant number of children (11537) aged 6–59.9 months, we observed that in our study population CCF were most utilized in urban settings (35).

More recently, a new form of packaging emerged, represented by compressible plastic bags equipped with a spout and a screw cap (pouch), with the infant sucking its content, making melas more easily administered, whether at home or on-the-go (36). Besides this major advantage, this form of presentation delays or hinders learning to eat from a spoon or “finger food” (37). A cross-sectional study by Haszard et al. revealed that a substantial proportion of infants, specifically 45.3%, consumed infant food pouches on at least one recall day. This finding underscores the prevalence of commercial infant food pouch usage in contemporary infant feeding practices. The high percentage of infants exposed to food pouches supports with broader tendencies observed in other developed countries, reflecting the growing popularity of these convenient feeding options (38).

We identified that 20% of the infants were never exposed to CCF products, while among the 80% consumers are offered a CCF product at least 1 day/week, supporting the available data. A study by Maslin et al. on CCF in developed countries revealed significant usage patterns in France. The majority of French parents (63%) incorporate commercially prepared baby foods into their infants’ diets 4–7 days per week (39). Similarly, The German DONALD (Dortmund Nutritional and Anthropometric Longitudinally Designed) study revealed that approximately 60% of complementary foods consumed by infants during the weaning period were commercially prepared. Comparable to our findings, only a small proportion of parents (24%) refrain entirely from using these products (40). Supplementary data arise from the United States indicating a high prevalence of CCF products consumption among infants aged 4–12 months, with estimates ranging from 73 to 95% (41). A cross-sectional study conducted by Hurley et al. on infants receiving benefits from the Special Supplemental Nutrition Program for Women, Infants, and Children in Maryland revealed significant insights into CCF consumption patterns. The study, which utilized a 24-h dietary recall methodology, found that among infants aged 6 to 12 months, 81% had consumed CCF within the past day (42).

The World Health Organization (WHO) defines marketing as “any form of commercial communication or message that is designed to, or has the effect of, increasing recognition, appeal and/or consumption of particular products and services” (43) Regarding the product awareness, a study conducted in Bangkok, Thailand revealed that approximately 90% of mothers reported exposure to at least one form of baby food marketing in the preceding 6 months, with electronic media being the primary source (44), consistent with our observation regarding that product awareness derived from advertising. The decision-making process for CCF adoption appears multifactorial. Advertising (36.7%) and social media (20%) were prominent initial sources of product awareness, often preceding recommendations from healthcare providers (11.6%). While advice from medical personnel was highly trusted when sought, peer influence and family tradition also carried substantial weight for many families. Breastmilk substitute (BMS) companies employ advanced digital marketing tactics—including targeted emails, influencer partnerships, webinars, and social media—to promote their products. They use emotional messaging and health claims to build parental trust and encourage formula and baby food use, which can undermine breastfeeding. Many parents trust these advertisements and view the products as safe and nutritious, despite misleading information (45). Additionally, interactions with healthcare professionals—whether through direct endorsements, participation in industry-sponsored events, or the dissemination of educational materials—contribute significantly to parental perceptions and acceptance of CCFs, although conflicts of interest may biase the objectivity of these recommendations (7).

Parental educational emerges as a significant predictor of CCF utilization, as evidenced by a multinational report from the European Union Childhood Obesity Project in 5 countries: Germany, Belgium, Italy, Poland and Spain. The findings indicate a proportion of 95% of infants consuming CCF at the age of 9 months while 68% of children were still CCF-fed at 24 months of age and suggest an inverse relationship between familial educational level and CCF consumption, with middle- and high-level parental education associated with reduced CCF usage (46).

Our research, however, did not reveal a statistically significant correlation between overall CCF consumption and mother educational level or family’s financial status, similar to the observations by Reidy et al. (47). When they used 24-h dietary recall data for 505 infants form The Feeding Infants and Toddlers Study (FITS) to describe CCF feeding pattern, the authors failed to identify differences regarding income and education between consumers and non CCF consumers. However, we identified one distinct association between the consumption of a specific CCF subtype, pseudocereals, and higher levels of maternal education. This finding aligns with the growing recognition of pseudocereals as nutritionally valuable alternatives in feeding practices (48–50). Other results from a study that comprised mothers of 6–23 months-old infants in SouthWest Ethiopia indicate the fact that higher wealth status and maternal employment were associated with CCF consume, which was reported in 44.3% of the infants (51). Furthermore, results from a study in India, revealed the fact that CCF are consumed to a less extent, in only 15% of children and mostly by highest socioeconomic group (52). A recent document highlights that education and economic status influence infant feeding choices, including the use of CFFs. In low- and middle-income countries, higher income and wealth increase CBF use, while in high-income countries, higher education and income often lead to more breastfeeding and less reliance on CBFs. Costs, cultural norms, and maternal employment also affect these decisions (53).

We presume that in developing economies such as Ethiopia and India, the CCF market is progressively expanding. The growth in national income is indirectly and proportionally reflected in the consumption of this type of nutrition, with its primary advantage being convenience. This assumption comes in line with broader economic trends observed in developing nations, where increased income often correlates with shifts in consumer behavior toward more convenient food options, likely because of changing lifestyles, urbanization, and increasing workforce participation of parents. This is in contrast to developed nations, where more extensive knowledge and trend toward natural products is part of a broader shift in consumer behavior, reflecting changing attitudes toward health, sustainability, and product authenticity. However, it is important to note that these trends are not uniformly distributed across all countries and may vary based on specific cultural, economic, and social factors within each nation.

Consistent with the European Union Childhood Obesity Project (46) which identified the group of cereals and pastas (including also biscuits, rusks) being most prevalent CCF during the beginning of CF, in our study we observed that flour-based products were the first administered CCF and furthermore, cereals, biscuits and pasta along with dairy-based products as the most consumed CCF products, followed by fruit purées and vegetable/vegetable-meat combination jars.

The elevated consumption of cereals and biscuits in our country can be attributed to historical infant feeding practices that predated the widespread availability of CCF and the current level of accessible information. During this earlier period, CF typically commenced with grated apple and biscuits, while milk-based semolina porridge served as a frequently utilized equivalent to traditional infant gruel in the diet of infants and young children. This historical context has likely shaped contemporary dietary preferences and consumption patterns. The cultural familiarity with cereals and biscuits as early CFs may have contributed to their continued popularity in the Romanian market.

Moreover, the same report (46) stated that dairy-based products were the most consumed CCF after the first year of life. In our study population we identified that this is the most offered CCF product in infants, but our study did not aim to extend research beyond the age of 1 year. We believe this phenomenon is a consequence of the Romanian market featuring several products of this type, packaged in colorful containers, which are based on cottage cheese and fruit puree. This combination is frequently used in CF meals for infants, mirroring the homemade preparation of cottage cheese (made by adding lactic calcium, lemon juice, or whey to milk) mixed with fruit purée, being the ready-to-eat alternative to homemade ones, attractive to both children and parents.

In our study, we identified a distinct preference for specific vegetables and fruits at an individual level. Surprisingly for our country, sweet potato emerged as the preferred vegetable in CCF products, followed by more traditionally choices like carrots and zucchini. Sweet potatoes possess notable nutritional benefits, particularly due to their high content of beta-carotene, anthocyanins, and lutein (54). The growing recognition of these nutritional advantages by parents is indirectly reflected in the remarkable 24-fold increase in sweet potato imports over the past decade (55). This surge in popularity is further supported by recent domestic cultivation efforts in Romania, potentially leading to a shift away from traditional vegetables like carrots, white potatoes, celery, and parsnips in complementary feeding practices (56). The use of carrots as one of the primary vegetable ingredient in infant CFs is a well-known practice as was reported in previous studies (57). Similarly to our findings are the results reported by Reidy et al. who identified sweet potatoes and carrots as the preferred ingredient in CCF products among 505 infants from The Feeding Infants and Toddlers Study (47).

Among fruits, apples and bananas were the most frequently utilized fruits in our cohort, similar to the reports of Bernal et al., who identified apples and bananas as the primary fruits in both homemade and commercially prepared products (58).

This selection pattern for vegetables and fruits may be attributed to several factors, including the year-round availability and relative affordability of these ingredients. Notably, sweet-tasting carrots and zucchini are common components in vegetable-based CCF, while apples and bananas frequently feature in fruit-based products (57, 59). A study conducted in the United States examining the variety and composition of vegetable-based CCF products found that red and orange vegetables, such as sweet potatoes and carrots, are most frequently used as primary ingredients in vegetable jars. These vegetables are often included in mixed-vegetable combinations, reflecting their popularity in CCF formulations. This combination pattern aligns with the broader preference for sweet-tasting vegetables in commercial products, which are more readily accepted by infants compared to bitter-tasting options (60).

Recent data from the Ministry of Agriculture on fish consumption in Romania reveals a national consumption rate of 88%, with the highest prevalence in the 35–64 age group and the lowest (24%) among 18–24-year-olds (61). Our findings, however, indicate that fish was the least frequently administered type of meat in infant diets. This discrepancy may be attributed to the age demographics of our study population, as mothers aged 20–30 years might align with the lower fish consumption patterns observed in the younger adult cohort of the national report. These results are consistent with previous research, such as the study by Mesch et al., which examined food variety in commercial and homemade complementary meals for infants in Germany. The observed low fish consumption in infant diets may reflect broader trends in dietary practices among younger parents, potentially influenced by factors such as nutritional knowledge, cultural preferences, or concerns about mercury content in fish (62).

Closely to our observations, the authors of a study that evaluated fish and rape seed oil in infants and 985 mothers in Germany, reported that only one-fourth of infants meet their recommended fish intake (at least once per-week) (63). On the contrary, reports from other countries indicate a higher fish consume in infancy (64, 65).

The ingredient preference pattern we observed is likely influenced, in part, by the fact that apples, bananas, carrots, and chicken are the most prevalent ingredients in CCFs, as described in a study analyzing the CCF market in Germany (66).

Very recent data comes from Carrillo et al. which aimed to evaluate the reason behind choosing commercial baby food purées. The authors identified that chicken and vegetables flavors like potato, onion, peas and legumes were associated with infants’ acceptance while fish, tomatoes and acidic flavors were negatively associated with acceptance. Moreover, textures like sandiness and stickiness were liked whereas smoothness was disliked (8).

A recent Italian study comparing commercial baby foods (CCF) and homemade alternatives revealed several advantages of CCF. The research found that CCF exhibited higher energy density and a more favorable macronutrient ratio. Moreover, CCF demonstrated superior safety profiles in terms of microbiological, pesticide residue, and mycotoxin contamination levels. Notably, the sodium content in CCF remained within recommended ranges. These findings suggest that commercially produced infant foods may offer certain nutritional and safety benefits over their homemade counterparts (67). Moreover, Houlihan et al. conducted a comprehensive analysis of heavy metal contamination in both CCF and homemade infant food preparations. Their investigation revealed no significant difference in heavy metal content between the two categories. The researchers concluded that homemade baby foods do not consistently demonstrate lower levels of toxic heavy metals compared to their commercially produced counterpart (68).

However, it’s important to note that the nutritional content and safety of both homemade and commercial baby foods can vary significantly depending on preparation methods, ingredient selection, and quality control measures (58, 67). Furthermore, CCF consumption is associated with greater quantity (grams) and variety of vegetables and fruits in the diet (42, 47). This may be attributed to the practice of “batch” preparation in homemade baby food, where larger quantities are typically made and offered over several consecutive days. This approach to home cooking for infants often results in repeated exposure to the same food items within a short timeframe, reflecting the practical considerations of meal planning and food storage for busy parents (39). This is concordance with the findings in our study. The main motivations for CCF use included good palatability (41.7%), nutritional value (16.7%), and product diversity (18.3%). Parents frequently cited the wide mixture of flavors and ingredients as attractive, facilitating exposure to new foods and textures. Parental motivations are nuanced and tied to socioeconomic status, with more educated or affluent families potentially being more attentive to perceived health benefits and trends (such as pseudocereals). Hollinrake et al. found that 95% of UK parents surveyed cited “convenience, time saving, and baby’s perceived enjoyment of products.” as a key motivation for purchasing commercial baby foods (69). Anxiety over food preparation, food safety, convenience, cost effectiveness were described by Isaacs et al. as advantages of CCF use (70). In a very recent comprehensive review CFFs are widely perceived by parents and caregivers as convenient, safe, and nutritionally beneficial options for their infants. Their ready-to-use formats save time and effort, making them appealing for busy families. Marketing claims emphasizing health benefits, such as “no added sugar” and developmental support, further enhance their attractiveness. However, despite these perceived advantages, CFFs often contain high levels of sugars, lack textural variety, and use misleading promotional messages that exploit parental concerns (71).

Furthermore, a qualitative study by Maslin et al. in the United Kingdom examined maternal perceptions and usage patterns of commercial infant foods. The research revealed that 45% of mothers with 8–10-month-old infants use CCF daily. Mothers generally viewed these products as convenient and safe, with some considering them superior to homemade options. While there were minor concerns about ingredient transparency, few mothers expressed worries about nutritional quality or allergen content (34). In a more recent survey conducted in Germany, in 2024, Hassig-Wegmann et al. evaluated the parental perception about CCF and analyzed the consumption patterns. The study revealed that 29% of participants reported using CCF daily, 13% consumed it almost daily, 20% used it several times a week, while 14% indicated that they never incorporated CCF into their infants’ diets (7), similar to our observation.

We found the principal advantage associated with CCF products their convenience as reported previously, while counterintuitive, long shell life was perceived as one of the disadvantages, contrary to reports from literature (34). Interestingly, the concern about “extended shelf-life” contrasts with literature promoting the safety of modern packaging. This gap between perception and laboratory-tested quality suggests that communication strategies about product composition and safety may need improvement. Several parents equated “long shelf-life” with artificial preservatives, even when products were actually free from such additives (7).

Regarding the study limitations, we primarily report that the population selection may be biased, on one hand, by the age of enrolled patients, given that younger ages are more frequently hospitalized, thus affecting the CCF consumer profile through age. On the other hand, socio-economic status and education level may also play a role, as limited resources at home (financial, social, intellectual) can contribute to the decision for hospitalization. Furthermore, in our hospital, admitted patients are mainly from Bucharest and surroundings counties. This renders the assessment of profiles associated with CCF utilization challenging for different regions or even ethnic groups residing within our country’s territory. Finally, we acknowledge that the sample size is relatively small in number given the nature of the research, limiting our ability to draw robust conclusions and confines our ability to extrapolate the results in the general population.

In contrast with the large literature on breastfeeding and complementary feeding practices from an overall perspective that has been reported globally, including our country, less attention has been paid to assessing CCF products’ use for a particular population. To our knowledge, this is the first study aimed at evaluating the specific trends of CCF use in Romania, while also outlining the consumer profile for these types of foods. This study contributes to a more precise understanding of the evolutionary tendencies in infant feeding, indirectly contributing to the characterization of Romania as a country converging progressively with ‘Westernization.” We believe our work could serve as a starting point for a multi-center evaluation, in non-hospitalized settings, that would more precisely delineate the characteristics of this feeding framework.

## 5 Conclusion

This study provides valuable insights into a dietary behavior that has not yet been investigated in our country. However, its most significant contribution lies not in this aspect alone, but in its potential to enhance our understanding of nutritional dynamics during a crucial developmental period. This research is particularly relevant given the potential influence of early feeding practices on the emergence of various health conditions later in life, a concept that has gained increasing attention in recent years, serving as a starting point for future investigations.

## Data Availability

The raw data supporting the conclusions of this article will be made available by the authors, without undue reservation.
